# Evaluation of Oral Health in Early Stages of Chronic Kidney Disease

**DOI:** 10.3390/dj14100646

**Published:** 2026-10-07

**Authors:** Sylvain Catros, Johan Samot, Virginie Chuy, Eléonore Tron, Fideline Collin, Antoine Bénard, Claire Rigothier

**Affiliations:** 1 Department of Oral Surgery, Centre Hospitalier Universitaire de Bordeaux, F-33076 Bordeaux, Francejohan.samot@u-bordeaux.fr (J.S.); 2 Laboratoire de Bioingénierie Tissulaire, Institut National de la Santé et de la Recherche Médicale, University of Bordeaux, U1026, F-33000 Bordeaux, France; 3 Bordeaux Sciences Agro, Bordeaux INP, INRAE, University of Bordeaux, UMR 1366, OENO, ISVV, F-33140 Villenave d’Ornon, France; 4 Pôle de Médecine et Chirurgie Bucco-Dentaire, Centre Hospitalier Universitaire de Bordeaux, F-33076 Bordeaux, France; 5 Bordeaux Population Health, Institut National de la Santé et de la Recherche Médicale, University of Bordeaux, U1219, F-33000 Bordeaux, France; 6 Clinical Epidemiology Unit (USMR), Pôle de Santé Publique, Centre Hospitalier Universitaire de Bordeaux, F-33076 Bordeaux, France; 7 Department of Clinical Research and Innovation, Centre Hospitalier Universitaire de Bordeaux, F-33076 Bordeaux, France; 8 Unit of Nephrology, Service de Néphrologie, Dialyse, Transplantation et Aphérèses, Centre Hospitalier Universitaire de Bordeaux, F-33076 Bordeaux, France

**Keywords:** chronic kidney disease, oral health, periodontal disease, nutrition, inflammation, diagnosis, oral

## Abstract

**Background/Objectives:** Oral health may be affected by chronic kidney disease (CKD) through mechanisms including uremic toxin accumulation, immune dysregulation, and treatment-related factors, although these relationships remain poorly understood. This study aimed to describe oral health according to CKD stages (2–5) and to explore its association with disease progression, as well as one-year changes in oral, renal, and inflammatory parameters and oral health–related quality of life. **Methods:** The NephroDent study is a prospective cohort study including patients with non-dialysis CKD stages 2 to 5. Oral, clinical, and biological parameters and quality of life were assessed at baseline and after one year. Baseline characteristics were compared across CKD stages, and changes were analyzed according to self-reported dental care. **Results:** Eighty-seven patients were included. Oral health impairment was observed across all CKD stages, including stages 2 and 3, with substantial periodontal treatment needs and periodontal pockets ≥ 6 mm. Dental and periodontal parameters varied across stages, with some indicators showing numerically poorer values in stages 4–5, but no significant differences were observed between CKD stages. Over one year, changes in oral health indices, renal function (eGFR), inflammatory markers, and quality of life did not differ significantly according to dental care. **Conclusions:** Oral health impairment was observed across all CKD stages, including early disease, despite preserved quality of life. No significant short-term association was found between dental care and changes in oral or systemic parameters. Further studies are needed to clarify the role of oral health in CKD progression.

## 1. Introduction

Chronic kidney disease (CKD) is a progressive condition characterized by a gradual decline in renal function, most commonly caused by diabetes, hypertension, or glomerulonephritis. CKD is classified into five stages, ranging from mild impairment (stages 1–2) to end-stage renal disease (stage 5), which requires dialysis or kidney transplantation. CKD is associated with reduced health-related quality of life and increased risk of premature mortality, affecting approximately 11% of adults in high-income countries, with prevalence increasing with age [1,2].

Renal dysfunction leads to the accumulation of uremic toxins and alterations in fluid, electrolyte, acid-base, and hormonal homeostasis. These systemic changes may contribute to oral health impairment through multiple pathways, including inflammatory dysregulation, salivary alterations, and treatment-related factors [3,4,5]. In CKD patients, periodontal disease, increased calculus formation, and alterations of oral hard tissues have been reported, particularly in advanced stages [6,7,8]. Salivary changes, including reduced buffering capacity and xerostomia, may further contribute to increased susceptibility to dental caries and oral infections [9,10]. Periodontitis is also more prevalent in CKD populations and may contribute to systemic inflammation, which is itself a recognized factor in CKD progression [11,12]. However, the available evidence does not clearly establish when specific oral alterations emerge or how they evolve across CKD stages.

Most previous studies have focused on patients with end-stage renal disease or those undergoing dialysis, whereas early-stage CKD remains less investigated despite representing the majority of the CKD population [12,13,14,15]. In addition, the relationship between oral health status and CKD progression remains incompletely understood, and current dental management in CKD patients is largely similar to that in the general population, without specific guidelines. A better characterization of oral health across CKD stages and of its evolution during follow-up may therefore help identify patients who could benefit from closer dental monitoring and provide a basis for future interventional studies.

We hypothesized that oral health impairment is present from early CKD stages and may worsen with disease progression. We also explored whether dental care exposure during follow-up was associated with changes in oral, renal, inflammatory, and quality of life parameters.

Therefore, the aim of this prospective study was to describe oral health according to CKD stage (stages 2–5) and to examine its association with renal function and systemic parameters. In addition, we assessed longitudinal changes in oral, renal, inflammatory, and oral-health-related quality of life outcomes over a one-year follow-up according to dental care exposure.

## 2. Materials and Methods

### 2.1. Study Population

The NephroDent study was a 1-year prospective cohort study. All participants were recruited from the Nephrology Unit of the University Hospital of Bordeaux (France) between September 2014 and July 2016. Eligible participants were adults (>18 years old) with non-dialysis chronic kidney disease (CKD) stages 2 to 5 who had not received dental care within the previous 6 months. Pregnant women and patients under legal guardianship were excluded.

The study was conducted in full accordance with ethical principles, including the Declaration of Helsinki (2002 version). Written informed consent was obtained from all participants prior to inclusion. The study protocol was independently reviewed and approved by the French ethics committee “CPP Sud-Ouest et Outre-Mer III” on 27 November 2013 (No. 2013-102). The study was registered at ClinicalTrials.gov (identifier: NCT02114281). We confirm that the study was conducted and reported in accordance with the STROBE guidelines.

To minimize the burden on participants, all parameters were collected during routine follow-up visits in the Nephrology Unit.

### 2.2. Oral Health Assessment

A comprehensive oral examination was performed by two calibrated senior oral surgeons who were blinded to the patients’ CKD stage at baseline (M0), 6 months (M6), and 12 months (M12). Prior to study initiation, the examiners underwent a calibration procedure based on the research protocol, including joint review of five panoramic radiographs and assessment of five patients to standardize the clinical assessment. Several validated indices were recorded to assess oral hygiene as well as the condition of the teeth, periodontium, and oral mucosa.

Oral hygiene was assessed using the Simplified Oral Hygiene Index (OHI-S), which ranges from 0 to 6 and quantifies dental plaque and calculus on six predefined index teeth [16]. The debris index and calculus index were calculated separately and then summed to obtain the individual OHI-S score. Scores were categorized as good (0–1.2), fair (1.3–3.0), or poor (3.1–6.0). When index teeth were missing, substitute teeth were used according to standard recommendations.

Dental status was assessed in accordance with World Health Organization (WHO) criteria. The Decayed, Missing, and Filled Teeth (DMFT) index (range: 0–28) was calculated based on the number of decayed, missing, and filled teeth [17]. In addition, the number of functioning teeth (FS-T index; defined as the number of sound and filled teeth) [18], the number of teeth indicated for extraction, and the number of functional tooth units (defined as pairs of opposing posterior natural or prosthetic teeth, excluding third molars) [19] were recorded.

Periodontal status was evaluated using the Community Periodontal Index of Treatment Needs (CPITN), using a WHO periodontal probe and standardized index teeth [20]. For this purpose, the dentition was divided into six sextants (upper right, upper anterior, upper left, lower left, lower anterior, and lower right), and one representative tooth per sextant was examined when present. Each sextant was assessed for bleeding on probing, calculus, and periodontal pocket depth. The highest score per sextant was recorded, and the overall CPITN score ranged from 0 (no treatment need) to 4 (need for complex periodontal treatment).

Finally, oral mucosal status was assessed in all participants through a systematic examination of the oral cavity, including the lips, commissures, vestibules, buccal mucosa, hard and soft palate, uvula, tonsils, tongue, floor of the mouth, and gingival tissues (free and attached gingiva, and gingival sulcus).

### 2.3. Renal Function and CKD Staging

Renal function was assessed as part of routine clinical care in the Nephrology Unit. The estimated glomerular filtration rate (eGFR) was calculated using the Modification of Diet in Renal Disease (MDRD) equation and used to classify patients according to chronic kidney disease (CKD) stages, based on the Kidney Disease Outcomes Quality Initiative (KDOQI) guidelines of the National Kidney Foundation [21].

CKD stages were defined as follows: stage 2 (eGFR 60–89 mL/min/1.73 m^2^), stage 3 (30–59 mL/min/1.73 m^2^), stage 4 (15–29 mL/min/1.73 m^2^), and stage 5 (<15 mL/min/1.73 m^2^).

### 2.4. Dental Care During the Study

Following the baseline dental examination, patients were informed of their oral health status and any indicated dental treatments. They were then advised to consult their general dental practitioner or, if they did not have one, to seek care at the Dental Department of the University Hospital of Bordeaux (France). Dental care received during the follow-up period was assessed at each study visit. Dental care was defined as any dental treatment or procedure performed by a dental professional during the follow-up period. Dental care was analyzed as a binary variable (yes/no).

### 2.5. Other Variables

Medical history and sociodemographic data (sex, age, and occupational category) were collected from medical records. Height and weight, used to calculate body mass index (BMI, kg/m^2^), were systematically recorded at baseline by the nephrologist. Information on alcohol consumption and smoking status (current, former, or never smoker) was obtained from medical records or, when unavailable, self-reported by patients during the baseline visit.

Medication use was systematically recorded at each study visit.

Inflammatory status was assessed as part of routine clinical follow-up using C-reactive protein (CRP), serum albumin, and prealbumin levels.

Oral health-related quality of life (OHRQoL) was assessed using the Oral Health Impact Profile-14 (OHIP-14) questionnaire [22]. The OHIP-14 consists of 14 items evaluating the impact of oral conditions on quality of life. Responses are scored on a 5-point Likert scale (0 = never; 1 = hardly ever; 2 = occasionally; 3 = fairly often; 4 = very often/every day). The total score ranges from 0 to 56 and is calculated by summing the item scores, with higher scores indicating a greater negative impact on quality of life. Domain scores range from 0 to 8.

### 2.6. Statistical Analyses

At the time the study was designed, no prior data were available to perform a formal sample size calculation. Therefore, the sample size was determined based on recruitment feasibility over an inclusion period not exceeding 18 months. Based on our experience, we anticipated enrolling approximately 30 patients in each CKD stage from stage 2 to stage 4, and 10 patients in stage 5, for a total of 100 patients. This sample size was considered sufficient to generate preliminary data on the potential impact of oral health management in this population.

Baseline sociodemographic, clinical, and oral characteristics were described according to CKD stage using means and standard deviations for continuous variables, and frequencies and percentages for categorical variables.

Changes between baseline (M0) and 12 months (M12) in oral health indices (OHI-S, FS-T, DMFT), renal function (eGFR), and inflammatory markers (CRP, albumin, and prealbumin), as well as OHIP-14 scores, were calculated. These changes were compared according to receipt of dental care during follow-up using Student’s *t* tests. Due to the small number of patients included in our sample, we did not perform multivariable statistical analysis. All analyses were conducted on available data.

All statistical tests were two-sided, and a *p* value < 0.05 was considered statistically significant. Statistical analyses were performed using SAS software (version 9.3 or later; SAS Institute Inc., Cary, NC, USA).

## 3. Results

### 3.1. Baseline Sociodemographic and Medical Characteristics

Among the 88 eligible patients, 87 were included in the present study (one patient was excluded after inclusion due to ineligibility). The study population was predominantly male (57.5%), with a mean age of 61.6 ± 14.3 years (Table 1).

At baseline, 23 patients (26%) had CKD stage 2, 34 (39%) stage 3, 23 (26%) stage 4, and 7 (8%) stage 5. Overall, most patients were retired or unemployed, and the mean body mass index (BMI) was 28.4 ± 5.9 kg/m^2^ (Table 1).

Approximately 10% of patients were current smokers in early CKD stages (stages 2 and 3), compared with 40% in advanced stages (stages 4 and 5). In contrast, former smokers were less frequent in advanced stages. Current alcohol consumption was reported in approximately one-third to one-half of participants, depending on CKD stage.

Treatments and comorbid conditions according to CKD stage are detailed in Appendix A Table A1 and Table A2.

Mean serum albumin and prealbumin levels were similar across CKD stages (overall mean values: 40.0 ± 5.1 g/L and 0.4 ± 0.06 g/L, respectively). In contrast, mean C-reactive protein (CRP) levels were higher in patients with CKD stage 2 (9.4 ± 26.9 mg/L) and stage 4 (8.4 ± 10.3 mg/L) (Table A3).

### 3.2. Baseline Oral Health Status and Quality of Life

Six patients were edentulous. Among patients with at least one evaluable sextant (n = 78), mean oral hygiene, as assessed by the OHI-S, was 1.0 ± 0.8 across the whole population and was similar across CKD stages.

Only one participant presented a clinically healthy periodontium. According to CPITN scores, more than 85% of patients required professional periodontal treatment, with the highest proportions observed in stages 3 and 4. Approximately 40% of patients (30% in stage 5) presented at least one periodontal pocket ≥ 6 mm.

The mean DMFT score was 15.2 ± 7.3 and increased with CKD stage, as did the numbers of decayed and missing teeth, in contrast to the number of filled teeth. Approximately one quarter of participants had at least one tooth indicated for extraction.

The mean number of functioning teeth (FS-T index) was 23.0 ± 5.9, and the mean number of functional tooth units was 4.2 ± 3.3. Both indices decreased in CKD stages 4 and 5.

Oral mucosal lesions, mainly keratosis, were observed in 54% of participants (Table 2 and Table A4).

At baseline, oral health–related quality of life (OHIP-14) scores were similar across CKD stages, indicating a generally low perceived impact of oral conditions on quality of life (Table A5).

### 3.3. Association Between Dental Care and the Evolution of Oral, Renal, Nutritional, and Inflammatory Parameters and of Quality of Life

After one year of follow-up, three patients died (two in CKD stage 2 and one in stage 4), and five patients were lost to follow-up (two in stage 3, two in stage 4, and one in stage 5). Among the 79 patients who completed the one-year follow-up, 57 received dental care after study inclusion, whereas 22 did not. Baseline demographic, renal (eGFR), inflammatory (CRP), oral health (OHI-S, DMFT, FS-T), and oral health-related quality of life (OHIP-14) characteristics were broadly comparable between patients who received dental care during follow-up and those who did not (Table A6). No significant differences were observed in age, sex, eGFR, CKD stage, CRP, OHI-S, DMFT, FS-T or OHIP-14.

Mean changes in OHI-S, FS-T, eGFR, serum albumin and prealbumin levels, and OHIP-14 scores between baseline and 12 months did not differ significantly according to whether or not dental care was received (Table 3).

The DMFT index increased in both groups, with a non-significantly greater increase observed in patients without dental care (+0.4 ± 1.0 vs. +1.2 ± 2.6, *p* = 0.158). Although not statistically significant, mean CRP levels decreased in patients who received dental care compared with those who did not (−3.5 ± 20.4 mg/L vs. +0.6 ± 9.9 mg/L, respectively; *p* = 0.410).

## 4. Discussion

The overall findings of this study indicate that oral health was impaired in patients with chronic kidney disease (CKD), regardless of disease stage, thereby confirming previous reports. Dental and periodontal parameters showed some numerical differences across CKD stages, but none of the major oral health indices differed significantly between stages. Most previous studies have focused on end-stage CKD or dialysis populations, and limited data are available on oral health in early-stage CKD.

The study population showed a relatively balanced distribution across CKD stages 2 to 4. Fewer patients with stage 5 CKD were included, as most patients at this stage followed in the Nephrology Unit were undergoing dialysis and therefore not eligible. Baseline sociodemographic and clinical characteristics of patients with stage 5 CKD were broadly comparable to those of other stages, although a lower proportion of active employment was observed, which is consistent with disease burden at advanced stages. Overall, body mass index was relatively preserved across the cohort.

Unexpectedly, patients with stage 5 CKD exhibited slightly higher prealbumin levels and lower inflammatory markers. This finding may reflect the characteristics of a single-center cohort with structured nutritional and medical follow-up, potentially leading to optimized clinical management. However, given the small number of patients in this subgroup, these results should be interpreted with caution. Tobacco and alcohol consumption remained frequent across all CKD stages, including advanced disease, and may contribute to poor oral health outcomes.

Our findings are in line with previous studies evaluating oral health in CKD populations. An Indian study including patients with CKD stages 2 to 5 reported poorer oral hygiene and periodontal status in more advanced stages [23]. By contrast, DMFT scores were higher compared with healthy controls but did not significantly differ across CKD stages. Similarly, a cross-sectional study reported a higher prevalence of severe periodontal disease in end-stage CKD compared with earlier stages [24]. However, longitudinal data remain scarce, and a 10-year follow-up study in a small Finnish cohort reported stable DMFT and periodontal indices despite progression of renal disease [25]. Taken together, these findings suggest that the relationship between CKD stage and oral health is not consistent across studies.

In our cohort, several dental and periodontal parameters showed numerical differences across CKD stages, but none of the major oral health indices differed significantly between stages. Over one year, we also found no significant association between dental care and changes in oral or renal outcomes. These findings should nevertheless be interpreted cautiously given the observational design and limited sample size. Larger longitudinal studies are needed to better clarify the relationship between CKD progression, oral health, and dental care.

The proportion of patients receiving dental care during follow-up did not differ across CKD stages. This may be explained by the relatively homogeneous characteristics of the study population and the systematic encouragement to seek dental care, either in private practice or at the hospital dental department, regardless of disease severity. In contrast, a large Japanese cohort study reported a decrease in dental attendance with increasing CKD severity, as well as a progressive decline in dental visits over time in high-risk populations [26].

Overall, the study population presented a relatively low inflammatory profile at baseline, likely reflecting good adherence to medical follow-up and optimized clinical management within a single university hospital. This may have limited the ability to detect differences between groups following dental care. Future studies should compare populations with more heterogeneous oral health status to better assess the potential systemic impact of oral interventions in CKD.

Regarding oral health–related quality of life, patients in our study reported relatively preserved scores at baseline and no significant changes over one year, regardless of CKD stage or dental care exposure. Previous studies have reported impaired oral health–related quality of life in CKD populations; however, comparisons remain difficult due to heterogeneity in assessment tools and study populations [27,28,29]. Notably, a cross-sectional study by Tabesh et al., using the OHIP-14 questionnaire, reported impaired oral health–related quality of life in patients with CKD, particularly in more advanced stages [29]. The relatively preserved quality of life observed in our cohort may reflect differences in disease severity, study design, or the characteristics of a well-monitored population.

This study has several limitations. The sample size was modest, and the number of patients with advanced CKD, particularly stage 5 CKD, was limited, reducing statistical power. This limited sample size may have impaired our ability to detect differences in oral health parameters across CKD stages; therefore, the absence of statistically significant differences should not be interpreted as evidence of the absence of clinically relevant associations. In addition, CKD stage 5 patients were underrepresented due to dialysis status, further limiting comparisons involving this group. Multivariable analyses were not performed because of the limited sample size and the relatively small number of patients within some CKD stages. Therefore, potential confounding by sociodemographic, behavioral, or clinical factors could not be fully assessed, and the observed associations should be interpreted with caution.

The observational design of the study also precludes any causal inference regarding the relationship between dental care and changes in oral, renal, inflammatory, or nutritional parameters during follow-up. Inter- and intra-examiner reliability of clinical oral assessments was not formally evaluated, although the use of validated indices likely minimized measurement bias. Furthermore, most clinical and biological data were collected as part of routine care, which may have reduced information bias. Finally, dental care exposure was self-reported, which may have introduced recall bias and potential misclassification. Although the type of dental care was documented during follow-up when reported by patients, these data were not systematically categorized for statistical analysis. Consequently, dental care was analyzed as a binary variable, which did not account for differences in the nature, frequency, or intensity of dental treatment. In addition, social desirability bias cannot be excluded, as some patients may have overreported dental care utilization, potentially attenuating differences between groups.

## 5. Conclusions

The relationship between oral health and CKD progression remains uncertain after this one-year monocentric study. Further studies with longer follow-up periods, larger sample sizes, and multicenter designs are needed to confirm and extend these findings.

At one year, no significant association was observed between dental care and the progression of oral health parameters or CKD-related outcomes. In addition, oral health–related quality of life remained stable over the follow-up period, regardless of CKD stage.

In patients with CKD, these findings highlight the need for further research to better understand the potential role of oral health management within comprehensive nephrological care strategies.

## Figures and Tables

**Table 1:** Sociodemographic and clinical characteristics at baseline of the 87 patients included in the study. NephroDent Study, Bordeaux (France), 2014–2016.

Baseline Sociodemographic and Clinical Characteristics	Chronic Kidney Disease				
	Overall N = 87	Stage 2 N = 23	Stage 3 N = 34	Stage 4 N = 23	Stage 5 N = 7
	N (%) or Mean (SD)	N (%) or Mean (SD)	N (%) or Mean (SD)	N (%) or Mean (SD)	N (%) or Mean (SD)
Men	50 (57.5%)	11 (47.8%)	22 (64.7%)	15 (65.2%)	2 (28.6%)
Age (years)	61.6 (14.3)	54.3 (16.2)	65.5 (12.5)	62.5 (14.2)	64.1 (8.2)
Occupational category:					
Executive or teacher	6 (6.9%)	3 (13.0%)	1 (2.9%)	2 (8.7%)	0 (0.0%)
Employee or worker	11 (12.6%)	5 (21.7%)	2 (5.9%)	3 (13.0%)	1 (14.3%)
Retired or unemployed	59 (67.8%)	12 (52.2%)	28 (82.4%)	13 (56.5%)	6 (85.7%)
Student and other	11 (12.6%)	3 (13.0%)	3 (8.8%)	5 (21.7%)	0 (0.0%)
BMI (kg/m^2^)	28.4 (5.9)	28.6 (5.9)	28.2 (5.9)	29.8 (6.3)	24.8 (4.2)
Tobacco consumption:					
Current smoker	19 (21.8%)	3 (13.0%)	4 (11.8%)	9 (39.1%)	3 (42.9%)
Ex-smoker	31 (36.9%)	10 (43.5%)	14 (41.2%)	6 (26.1%)	1 (14.3%)
Alcohol:					
Current alcohol consumption	34 (40.0%)	11 (47.8%)	10 (29.4%)	10 (43.5%)	3 (42.9%)
History of alcohol consumption	12 (19.4%)	1 (6.7%)	7 (26.9%)	4 (25.0%)	0 (0.0%)

BMI: body mass index; SD: standard deviation.

**Table 2:** Baseline oral health status according to the chronic kidney disease stage. NephroDent Study, Bordeaux (France), 2014–2016.

Baseline Oral Characteristics	Chronic Kidney Disease										
	Overall		Stage 2		Stage 3		Stage 4		Stage 5		*p*-Value
	N = 87		N = 23		N = 34		N = 23		N = 7		
	N (%) or Mean (SD)		N (%) or Mean (SD)		N (%) or Mean (SD)		N (%) or Mean (SD)		N (%) or Mean (SD)		
**OHI-S index**	1.0	(0.8)	0.9	(0.6)	0.9	(0.9)	1.3	(1.0)	0.8	(0.6)	0.4481 *
**CPI**											0.108 ^§^
Health	1	(1.1%)	0	(0.0%)	0	(0.0%)	1	(4.8%)	0	(0.0%)	
Bleeding on probing	10	(11.5%)	4	(19.1%)	3	(10.0%)	1	(4.8%)	2	(28.6%)	
Plaque/calculus	13	(14.9%)	6	(28.6%)	1	(3.3%)	6	(28.6%)	0	(0.0%)	
Pocket(s) 4–5 mm	19	(21.8%)	2	(9.5%)	11	(36.7%)	4	(19.1%)	2	(28.6%)	
Pocket(s) ≥ 6 mm	35	(40.2%)	9	(42.9%)	15	(50.0%)	9	(42.9%)	2	(28.6%)	
**Periodontal treatment needs**											0.602 ^§^
No need for improving oral hygiene	11	(14.1%)	4	(19.1%)	3	(10.0%)	2	(9.5%)	2	(33.3%)	
Need for professional dental care	67	(85.9%)	17	(81.0%)	27	(90.0%)	19	(90.5%)	4	(66.6%)	
**DMFT index**	15.2	(7.3)	13.6	(7.3)	15.1	(7.3)	16.7	(7.2)	16.0	(7.5)	0.555 ^§^
**No. of decayed teeth**	1.7	(2.9)	1.4	(2.1)	1.5	(2.8)	2.3	(3.5)	2.1	(3.4)	
**No. of missing teeth**	7.7	(8.2)	6.9	(8.2)	7.2	(8.7)	8.8	(7.6)	9.9	(8.3)	
**No. of filled teeth**	5.7	(5.1)	5.3	(4.8)	6.4	(5.6)	5.5	(4.8)	4.0	(4.0)	
**Teeth to be extracted ≥1**	24	(27.5%)	6	(26.1%)	8	(23.5%)	8	(34.8%)	2	(28.6%)	
**FS-T index**	23.0	(5.9)	23.3	(5.2)	24.0	(5.4)	21.7	(7.0)	21.4	(5.8)	0.46 *
**No. of functional units**	4.2	(3.3)	4.5	(3.2)	4.6	(3.3)	3.5	(3.2)	3.1	(3.4)	
**Mucosal lesions**	47	(54%)	10	(43.5%)	19	(55.9%)	16	(69.6%)	2	(28.6%)	

CPI: community periodontal index; DMFT: decayed missing filled per tooth; FS-T: number of functional teeth; OHI-S: simplified oral hygiene index; SD: standard deviation. * One-way analysis of variance (ANOVA); ^§^ Fisher exact test.

**Table 3:** Comparison of mean differences between baseline and one-year follow-up in oral, renal, nutritional and inflammatory parameters and in quality of life according to dental care received during the year. NephroDent Study, Bordeaux (France), 2014–2016.

Variables	Overall N = 79			Dental Care N = 57			No Dental Care N = 22			*p*-Value *
	N	Mean	(SD)	N	Mean	(SD)	N	Mean	(SD)	
OHI-S index	70	0.0	(0.6)	53	0.0	(0.6)	17	0.2	(0.5)	0.124
DMFT index	77	1.0	(2.3)	55	0.4	(1.0)	22	1.2	(2.6)	0.158
FS-T index	77	0.1	(2.0)	55	−0.2	(1.4)	22	0.2	(2.2)	0.422
eGFR (mL/min/1.73 m^2^)	77	−1.9	(9.0)	55	−2.2	(9.6)	22	−1.3	(7.6)	0.709
Albumin (g/L)	75	0.3	(4.6)	53	0.7	(4.8)	22	−0.8	(3.8)	0.204
Pre-albumin (g/L)	63	−0.1	(0.5)	42	−0.1	(0.7)	21	0.0	(0.1)	0.526
CRP (mg/L)	64	−2.2	(17.8)	44	−3.5	(20.4)	20	0.6	(9.9)	0.410
OHIP-14	77	−0.4	(3.9)	55	−0.9	(3.7)	22	1.0	(4.4)	0.063

CRP: C-reactive protein; DMFT: decayed missing filled per tooth; FS-T: number of functional teeth; eGFR: glomerular filtration rate; OHIP-14: Oral Health Impact Profile-14; OHI-S: simplified oral hygiene index; SD: standard deviation. * Student’s *t* test.

## Data Availability

All data generated or analyzed during this study are included in this article. The underlying raw data are available from the corresponding author upon reasonable request.

## Abbreviations

The following abbreviations are used in this manuscript:

BMI	Body Mass Index
CKD	Chronic Kidney Disease
CPITN	Community Periodontal Index of Treatment Needs
CPP	Comité de Protection des Personnes
CRP	C-reactive protein
DMFT	Decayed Missing Filled per Tooth
eGFR	Estimated Glomerular Filtration Rate
FS-T	Number of functioning teeth
M0, M6, M12	Month 0 (Baseline), Month 6, Month 12
MDRD	Modification of Diet in Renal Disease
OHIP-14	Oral Health Impact Profile-14 questions
OHRQoL	Oral health–related quality of life
OHI-S	Simplified Oral Hygiene Index
RAAS	Renin–Angiotensin–Aldosterone System
WHO	World Health Organization

## Appendix A

**Table A1:** Treatment at baseline of the 87 patients included in the study. NephroDent Study, Bordeaux (France), 2014–2016.

Treatments	Chronic Kidney Disease				
	Overall N = 87	Stage 2 N = 23	Stage 3 N = 34	Stage 4 N = 23	Stage 5 N = 7
	N (%)	N (%)	N (%)	N (%)	N (%)
Anxiolytics	42 (48,3)	8 (34,8)	17 (50,0)	13 (56,5)	4 (57,1)
Corticoids	19 (21,8)	5 (21,7)	11 (32,4)	3 (13,0)	0 (0,0)
Vitamin D	87 (100,0)	23 (100,0)	34 (100,0)	23 (100,0)	7 (100,0)
Biphosphonates	2 (2,3)	1 (4,3)	1 (2,9)	0 (0,0)	0 (0,0)
Immunosuppressors	24 (27,6)	6 (26,1)	11 (32,4)	7 (30,4)	0 (0,0)
Calcium channel blockers	38 (43,7)	5 (21,7)	15 (44,1)	11 (47,8)	7 (100,0)
Anticoagulant	14 (16,1)	1 (4,3)	8 (23,5)	4 (17,4)	1 (14,3)
Antiplatelet	42 (48,3)	10 (43,5)	15 (44,1)	13 (56,5)	4 (57,1)
RAAS inhibitors	80 (92,0)	20 (87,0)	34 (100,0)	20 (87,0)	6 (85,7)

**Table A2:** Pathological associated conditions at baseline of the 87 patients included in the study. NephroDent Study, Bordeaux (France), 2014–2016.

	Chronic Kidney Disease				
	Overall N = 87	Stage 2 N = 23	Stage 3 N = 34	Stage 4 N = 23	Stage 5 N = 7
	N (%)	N (%)	N (%)	N (%)	N (%)
Diabetic nephropathy	16 (18,4)	2 (8,7)	7 (20,6)	6 (26,1)	1 (14,3)
Endocrine diseases	59 (67,8)	14 (60,9)	29 (85,3)	14 (60,9)	2 (28,6)
Inflammatory diseases	13 (14,9)	6 (26,1)	3 (8,8)	3 (13,0)	1 (14,3)
Cancer	23 (26,4)	5 (21,7)	13 (38,2)	4 (17,4)	1 (14,3)
Hematologic diseases	18 (20,7)	1 (4,3)	5 (14,7)	9 (39,1)	3 (42,9)

**Table A3:** Biological characteristics at baseline of the 87 patients included in the study. NephroDent Study, Bordeaux (France), 2014–2016.

Biological Characteristics	Chronic Kidney Disease				
	Overall N = 87	Stage 2 N = 23	Stage 3 N = 34	Stage 4 N = 23	Stage 5 N = 7
	Mean (SD)	Mean (SD)	Mean (SD)	Mean (SD)	Mean (SD)
Albumin (g/L)	40,0 (5,1)	40,4 (4,0)	40,3 (3,9)	38,6 (7,6)	41,8 (3,1)
Pre-albumin (g/L)	0,4 (0,6)	0,3 (0,1)	0,3 (0,1)	0,4 (0,5)	1,0 (1,6)
CRP (mg/L)	7,5 (15,9)	9,4 (26,9)	6,1 (9,5)	8,4 (10,3)	5,0 (5,6)

SD: standard deviation; CRP: C-reactive protein.

**Table A4:** Oral mucosa lesions at baseline. NephroDent Study, Bordeaux (France), 2014–2016.

		Chronic Kidney Disease									
		Overall N = 87		Stage 2 N = 23		Stage 3 N = 34		Stage 4 N = 23		Stage 5 N = 7	
		N	(%)	N	(%)	N	(%)	N	(%)	N	(%)
**Lips**											
	Keratosis	1	(1.3%)	1	(4.3%)						
	Nodule	1	(1.3%)			1	(2.9%)				
**Commissures**											
	Keratosis	1	(1.3%)					1	(4.3%)		
**Vestibule**											
	Keratosis	2	(2.5%)			2	(5.9%)				
	Pigmentation	1	(1.3%)			1	(2.9%)				
**Jugal mucosa**											
	Keratosis	3	(3.8%)			1	(2.9%)	2	(8.7%)		
	Pigmentation	5	(6.3%)	2	(8.7%)	1	(2.9%)	2	(8.7%)		
	Other	2	(2.5%)			2	(5.9%)				
**Hard palate, soft palate and uvula**											
	Pigmentation	4	(5.0%)			4	(11.8)				
	Erythema	2	(2.3%)			1	(2.9%)			1	(14.3)
	Pseudomembrane	1	(1.3%)					1	(4.3%)		
**Tongue**											
	Keratosis	2	(2.5%)	1	(4.3%)	1	(2.9%)				
	Nodule	2	(2.5%)			2	(5.9%)				
	Pigmentation	1	(1.3%)			1	(2.9%)				
	Erythema	2	(2.3%)					1	(4.3%)	1	(14.3%)
	Pseudomembrane	1	(1.3%)					1	(4.3%)		
	Atrophy	2	(2.5%)	1	(4.3%)			1	(4.3%)		
**Floor of the mouth**											
	Petechiae	1	(1.3%)			1	(2.9%)				
**Gingival sulcus, free and attached gingiva**											
	Keratosis	6	(7.5%)	1	(4.3%)	4	(11.8%)	1	(4.3%)		
	Pigmentation	5	(6.3%)	1	(4.3%)	2	(5.9%)	2	(8.7%)		
	Erythema	5	(6.3%)			2	(5.9%)	3	(13.0%)		
	Oedema	11	(12.6%)	3	(13.0%)	4	(11.8%)	3	(13.0%)	1	(14.3%)

**Table A5:** Oral Health Impact Profile 14 (OHIP-14).

	Chronic Kidney Disease				
OHIP-14	Overall N = 87	Stage 2 N = 23	Stage 3 N = 34	Stage 4 N = 23	Stage 5 N = 7
	Mean (SD)	Mean (SD)	Mean (SD)	Mean (SD)	Mean (SD)
M0	5.7 (5.1)	5.4 (4.8)	6.1 (5.2)	4.7 (4.3)	7.4 (8)
M6	5.7 (5.5)	6.0 (5.8)	5.6 (5.8)	4.8 (4.2)	8.1 (7)
M12	5.0 (4.5)	5.6 (5.4)	5.2 (4.0)	4.3 (4.8)	3.9 (3.6)

OHIP-14: Oral Health Impact Profile; SD: standard deviation; M0: baseline; M6: 6 months visit; M12: 12 months visit.

**Table A6:** Comparison of baseline characteristics between patients who received dental care and those who did not.

Variables	Overall			Dental Care			No Dental Care			*p*-Value *
Age, *n* mean (SD)	79	60.8	(14.5)	57	60.9	(13.8)	22	60.5	(16.5)	0.905
eGFR, *n* mean (SD)	79	43.8	(23.0)	57	45.1	(22.9)	22	40.2	(23.3)	0.396
CRP, *n* median (IQR)	75	3	(1–7)	53	3	(1–7)	22	5	(1-10)	0.842
OHI-S index, *n* mean (SD)	70	1.0	(0.8)	53	1.0	(0.8)	17	0.8	(0.9)	0.452
DMFT index	79	15	(7.3)	57	14.2	(6.1)	22	17	(9.6)	0.1147
FS-T index	79	23.2	(5.4)	57	23.6	(4.5)	22	22.4	(7.2)	0.3705
OHIP-14, *n* mean (SD)	79	5.7	(5.0)	57	6.1	(5.3)	22	4.7	(4.1)	0.285
Male sex, *n* (%)		45	(57.0%)		34	(59.6%)		11	(50.0%)	0.459
CKD stage, *n* (%)										
2		22	(27.8%)		17	(29.8%)		5	(22.7%)	
3		30	(38.0%)		23	(40.4%)		7	(31.8%)	
4		20	(25.3%)		12	(21.1%)		8	(36.4%)	
5		7	(8.9%)		5	(8.8%)		2	(9.1%)	

CRP: C-reactive protein; DMFT: decayed missing filled per tooth; FS-T: number of functional teeth; eGFR: glomerular filtration rate; OHIP-14: Oral Health Impact Profile-14; OHI-S: simplified oral hygiene index; SD: standard deviation. * Student’s *t* test.
